# Nursing Students’ Attitudes Toward Technology: Multicenter Cross-Sectional Study

**DOI:** 10.2196/50297

**Published:** 2024-04-29

**Authors:** Ana Luiza Dallora, Ewa Kazimiera Andersson, Bruna Gregory Palm, Doris Bohman, Gunilla Björling, Ludmiła Marcinowicz, Louise Stjernberg, Peter Anderberg

**Affiliations:** 1 Department of Health Blekinge Institute of Technology Karlskrona Sweden; 2 Department of Health and Caring Sciences Linnaeus University Växjö Sweden; 3 Department of Mathematics and Natural Sciences Blekinge Institute of Technology Karlskrona Sweden; 4 Optentia Research Unit North West University Vanderbijlpark South Africa; 5 School of Health and Wellfare Jönköping University Jönköping Sweden; 6 Faculty of Nursing Kilimanjaro Christian Medical University College Moshi United Republic of Tanzania; 7 Department of Neurobiology, Care Sciences and Society Karolinska Institutet Stockholm Sweden; 8 Faculty of Health Sciences Medical University of Bialystok Białystok Poland; 9 Department of Care Science Malmö University Malmö Sweden; 10 Swedish Red Cross University Huddinge Sweden; 11 School of Health Sciences University of Skövde Skövde Sweden

**Keywords:** nursing education, technophilia, eHealth, technology anxiety, technology enthusiasm, mobile phone

## Abstract

**Background:**

The growing presence of digital technologies in health care requires the health workforce to have proficiency in subjects such as informatics. This has implications in the education of nursing students, as their preparedness to use these technologies in clinical situations is something that course administrators need to consider. Thus, students’ attitudes toward technology could be investigated to assess their needs regarding this proficiency.

**Objective:**

This study aims to investigate attitudes (enthusiasm and anxiety) toward technology among nursing students and to identify factors associated with those attitudes.

**Methods:**

Nursing students at 2 universities in Sweden and 1 university in Poland were invited to answer a questionnaire. Data about attitudes (anxiety and enthusiasm) toward technology, eHealth literacy, electronic device skills, and frequency of using electronic devices and sociodemographic data were collected. Descriptive statistics were used to characterize the data. The Spearman rank correlation coefficient and Mann-Whitney *U* test were used for statistical inferences.

**Results:**

In total, 646 students answered the questionnaire—342 (52.9%) from the Swedish sites and 304 (47.1%) from the Polish site. It was observed that the students’ technology enthusiasm (techEnthusiasm) was on the higher end of the Technophilia instrument (score range 1-5): 3.83 (SD 0.90), 3.62 (SD 0.94), and 4.04 (SD 0.78) for the whole sample, Swedish students, and Polish students, respectively. Technology anxiety (techAnxiety) was on the midrange of the Technophilia instrument: 2.48 (SD 0.96), 2.37 (SD 1), and 2.60 (SD 0.89) for the whole sample, Swedish students, and Polish students, respectively. Regarding techEnthusiasm among the nursing students, a negative correlation with age was found for the Swedish sample (*P*<.001; ρ_Swedish_=−0.201) who were generally older than the Polish sample, and positive correlations with the eHealth Literacy Scale score (*P*<.001; ρ_all_=0.265; ρ_Swedish_=0.190; ρ_Polish_=0.352) and with the perceived skill in using computer devices (*P*<.001; ρ_all_=0.360; ρ_Swedish_=0.341; ρ_Polish_=0.309) were found for the Swedish, Polish, and total samples. Regarding techAnxiety among the nursing students, a positive correlation with age was found in the Swedish sample (*P*<.001; ρ_Swedish_=0.184), and negative correlations with eHealth Literacy Scale score (*P*<.001; ρ_all_=−0.196; ρ_Swedish_=−0.262; ρ_Polish_=−0.133) and with the perceived skill in using computer devices (*P*<.001; ρ_all_=−0.209; ρ_Swedish_=−0.347; ρ_Polish_=−0.134) were found for the Swedish, Polish, and total samples and with the semester only for the Swedish sample (*P*<.001; ρ_Swedish_=−0.124). Gender differences were found regarding techAnxiety in the Swedish sample, with women exhibiting a higher mean score than men (2.451, SD 1.014 and 1.987, SD 0.854, respectively).

**Conclusions:**

This study highlights nursing students’ techEnthusiasm and techAnxiety, emphasizing correlations with various factors. With health care’s increasing reliance on technology, integrating health technology–related topics into education is crucial for future professionals to address health care challenges effectively.

**International Registered Report Identifier (IRRID):**

RR2-10.2196/14643

## Introduction

### Background

Health care costs have been growing faster than the economy for the past 17 years [1]. This upward trend is due to multifactorial causes related to the growth and aging of the population, increased prevalence of lifestyle-related noncommunicable diseases, increased prices of health services and pharmaceuticals, and the risk of global pandemics [2-4]. All these factors put high pressure on the health care systems, which have to deal with many challenges related to efficiency and productivity. The digitalization of the health care sector is strongly influencing the efforts to address health care challenges and involves the use of technologies such as information and communication technologies in health settings, which was later termed as *eHealth* [5].

The integration of eHealth in the health care sector points to greater use of technology to access health data, manage eHealth records, and engage in telehealth platforms, among others [6]. This is such an important topic that the European Commission issued the Digital Decade Policy Program targeting Europe’s digital transformation by 2030 [7]. This policy envisions, among other goals, the achievement of a digitally skilled population, highlighting the importance of a highly digitally skilled health care workforce and inspiring initiatives in different European countries. In the United States, a similar government initiative promotes the use of health technologies to improve the quality, safety, and efficiency of and reduce disparities in health care delivery [8]. The merging of health care workforce and digital technologies became so evident that informatics is outlined as one of the core competencies in the nursing profession: “use information and technology to communicate, manage knowledge, mitigate error and support decision making” [9]. Accordingly, it is also increasingly important for registered nurses to become proficient in this aspect.

Incorporation of health technologies into nursing education and the preparedness of the new students to use these in clinical scenarios and practice are highly important and a growing concern for program administrators, educators, researchers, policy makers, and employers [10]. This concern is valid because despite many students having grown up with technology ingrained in their everyday life, they still report low confidence, difficulties, and not-so-positive views about applying digital skills in clinical contexts [11-15]. Therefore, it is important to investigate the nursing students’ attitudes toward technologies, so that appropriate decisions can be made for educational purposes that might affect future patient care.

Many models assess user interaction with technology according to factors such as acceptance, motivation, adoption, adaptivity, and usability, which are known to play a role in technology use [16]. However, it is argued that both cognitive and emotional effectiveness affect behavior, and these are underlying factors that precede the specific, planned, and reasoned actions directed toward technology [17,18]. The concept of technophilia is a personality trait and a psychological construct that is related to a person’s enthusiasm or positive feelings toward technology use and the absence of anxiety or fears and doubts regarding technology [19], and it is a general quality that could potentially influence a wide range of aspects of technology use. Contrary to models tailored to specific organizational tasks, the investigation of technophilia could provide a better picture of the students’ needs regarding this proficiency.

### Objectives

This study comprises a multicenter, cross-sectional investigation of technophilia among nursing students that aimed to (1) establish the levels of technophilia among nursing students of 3 educational institutions in Sweden and Poland regarding their enthusiasm and anxiety and (2) identify factors that could be associated with the students’ technology enthusiasm (techEnthusiasm) and technology anxiety (techAnxiety).

## Methods

### Study Design

This study used a multicenter, cross-sectional design based on questionnaire data collected from nursing students in 3 different universities, in Sweden and Poland, in different stages in their education. The protocol for this study has been described previously [20]. This study adhered to the STROBE (Strengthening the Reporting of Observational studies in Epidemiology) guideline for cross-sectional studies (Multimedia Appendix 1).

### Setting

We collected data in the period between December 2019 and April 2020, using questionnaires administered to students enrolled in the nurse education programs of 3 universities: 2 in Sweden (Blekinge Institute of Technology [BTH] and Swedish Red Cross University [SRCU]) and 1 in Poland (Medical University of Bialystok [MUB]). The undergraduate nursing education of both countries adheres to the European Union requirements, which comprises 180 European Credit Transfer and Accumulation System (ECTS) credits at the university level [21,22]. The educational programs in both countries result in a professional degree (ie, a diploma) and an academic degree (ie, a bachelor’s degree), qualifying for a license as a registered nurse. At the time the study, the Swedish nursing education consisted of both theoretical and clinical practice courses—60% and 40% of the total curriculum, respectively. At the Polish institution, MUB, the nursing program consisted of 52% theoretical courses and 48% clinical practice courses. The students’ exposure to eHealth or health technology courses at the time of the data collection was as follows:

- At BTH, eHealth is covered in nursing subjects during the whole program and in two dedicated courses in the curriculum:

1. An eHealth introductory course is offered in the third semester to all students (4.5 ECTS), which was completed at the time of the data collection.
2. An optional course on digitalization and eHealth was offered in the fifth semester (7.5 ECTS). It was chosen by approximately one-third of the fifth-semester students and was ongoing at the time of the data collection.

- At SRCU, eHealth was also incorporated into nursing subjects during the whole program and 1 optional course (7.5 ECTS) in medical technology, digitalization, and eHealth was offered in the fifth semester. However, this course started 5 weeks after this study’s data collection.
- At MUB, eHealth was incorporated into nursing courses during the whole program.

### Participants and Data Collection Procedures

A convenience sample of undergraduate nursing students, enrolled at the bachelor of nursing program at BTH, SRCU, and MUB, was used in this study. Students from the first, third, and fifth semesters were eligible to participate in this study. These semesters were chosen to obtain a sample incorporating the beginning, middle, and end of nursing education, which comprises 6 semesters.

Data were collected using a paper-based questionnaire administered to all undergraduate students from the first, third, and fifth semesters of the participating universities by research members who had no educational connections to the students. This was done to minimize response bias.

### Questionnaire

The questionnaire was used to collect data about the participants’ sociodemographics, self-reported attitudes toward technology, eHealth literacy, perceived skills in using electronic devices, and frequency of using electronic devices.

#### Data on Attitudes Toward Technology (Technophilia Instrument)

The outcome measures of this study are the self-reported data on attitudes toward technology scored by the Technophilia instrument (TechPH) [19]. The TechPH comprises 6 questions to capture behaviors related to adaptation and use of a new technology, which were derived from the content analysis of relevant technophilia measures. It results in 2 numeric scores ranging from 1 (low) to 5 (high): techEnthusiasm and techAnxiety. The TechPH was originally developed for measuring older adults’ attitudes toward technology; however, published studies have already applied it on younger individuals, physicians, and dementia caretakers aged 18 to 44 years [23,24]. In this study, techEnthusiasm and techAnxiety have Cronbach α of 1 and 0.925, respectively, showing excellent internal consistency.

#### Sociodemographic Data

Sociodemographic data consisted of the participants’ age; gender; focus of high school studies (health or social care, technology, or other); and previous work experience (health or social care, technology, or other).

#### eHealth Literacy Data (eHealth Literacy Scale)

The eHealth literacy was scored using the eHealth Literacy Scale (eHEALS) instrument [25]. The eHEALS is a self-report tool consisting of 8 questions and has already been validated in many languages and diverse populations including undergraduate health professionals [25,26]. The eHEALS produces a score ranging from 1 (low eHealth literacy) to 5 (high eHealth literacy).

#### Data on Perceived Skill in and Frequency of Using Technological Devices

Perceived skills in using electronic devices, namely, computers or laptops, tablets, and smartphones, were rated using a Likert-type scale ranging from 1 (not knowledgeable at all) to 5 (very knowledgeable). The frequencies of using electronic devices were rated using a Likert-type scale ranging from 1 (several times daily) to 5 (never).

### Data Analysis

The descriptive statistics, namely, frequency, mean, and SD, were used to analyze the collected data. The Shapiro-Wilk test was used to assess data distribution. As the data were not normally distributed, nonparametric tests were used in the statistical analyses. Spearman rank correlation coefficient was used to measure the association among age, semester, perceived skills in using computers or laptops, and frequency of using electronic devices via the self-reported TechPH components—techEnthusiasm and techAnxiety. CIs were calculated to analyze the stability of the results. Mann-Whitney *U* test was used to assess gender differences regarding students’ enthusiasm and anxiety toward technology. Sensitivity analyses were performed by removing the outliers and revealed that the interpretations were unperturbed, showing that extreme data points did not impact the study outcomes. For all the analyzes, a significance level of .05 was used. Stratification was used; therefore, results are presented for the whole sample, Swedish students, and Polish students separately, to control for confounding. Entries with missing data were omitted from the analysis. The analyses were performed using R (version 1.4.1717; RStudio).

### Ethical Considerations

The study was conducted in accordance with the Declaration of Helsinki [27]. Participation in the study was voluntary. All participants were briefed about the study aims; that they could choose to not submit the questionnaire or submit it blank; and that by submitting the questionnaire, they would consent to participate in the study. All collected data were anonymous.

Permission to conduct the study was obtained from heads of the departments at all participating universities. In Poland, ethics approval was obtained from the ethics committee of Medical University of Bialystok (register number R-I-002/148/2017). In Sweden, the study did not require ethics approval according to the requirements of the Swedish Ethical Review Act 2003:460, 3-4§ [28], as the study did not explore sensitive personal data (eg, health, religion, political views, and ethnic heritage) or data relating to criminal offenses, did not involve physical intervention on the participants, and did not aim to affect the participants in any way or involve biological material.

## Results

### Sample Characteristics

In total, 646 students answered the questionnaire—342 (52.9%) from the Swedish sites and 304 (47.1%) from the Polish site. The response rates were 70.2% (646/920) for the whole sample, 63.1% (342/542) for the Swedish students, and 80.4% (304/378) for the Polish students. Nonresponders include students who decided not to submit the questionnaire or to submit it blank. None of the variables used in the analyses contained >5% of missing values.

**Figure 1:**
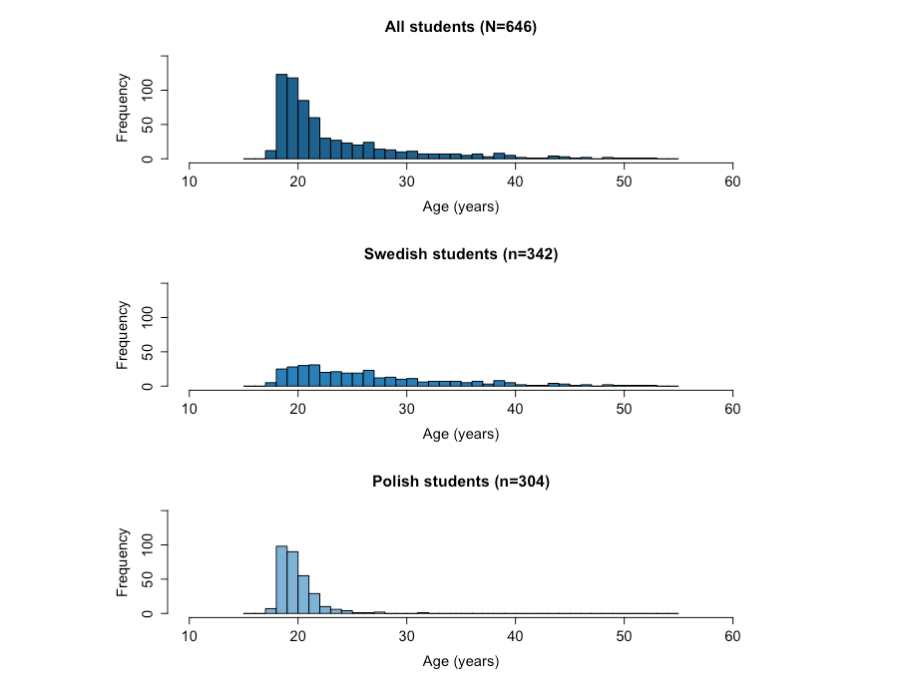
Distribution of ages in the whole sample, Swedish students, and Polish students.

The descriptive statistics are shown in Table 1, for the whole sample and for the Swedish and Polish students separately. Multimedia Appendix 2 shows the descriptive statistics along with the means and SDs for the techAnxiety and techEnthusiasm for each grouping shown in Table 1—for the whole sample, Swedish students, and Polish students separately. The mean age of the sample is 23.9 (SD 6.39) years, with the Swedish students being generally older and having a higher age variance (mean 27, SD 7.34 years) compared with the Polish students (mean 20.4, SD 1.72 years), as shown in Figure 1. While the Polish sample has a distribution that is more concentrated around the mean, the Swedish sample has a flatter distribution of ages. The sample was majorly composed of women students (555/646, 85.9%). Very few students had a high school focus on or previous work experience with technology before their nursing education. Overall, 50.3% (153/304) of the Polish students had a health and social care focus in high school, while this number was 23.9% (82/342) for the Swedish students. In terms of perceived skill in using electronic devices, most participants perceive themselves “knowledgeable” or “very knowledgeable” in all 3 categories: computers (479/646, 74.1%), smartphones (574/646, 88.9%), and tablets (388/646, 60.1%). Furthermore, 48.6% (314/646) of the participants answered that they use computers or laptops “several times daily” or “daily,” while this number reached 98.8% (638/646) for smartphones and 11.2% (72/646) for tablets. The students showed an overall high eHealth literacy, with 93.3% (603/646) scoring ≥3 points. The mean eHEALS scores for the overall, Polish, and Swedish samples were 3.95 (SD 0.75), 3.96 (SD 0.78), and 3.95 (SD 0.73), respectively, constituting high scores and showing an overall high perceived eHealth literacy.

**Table 1:** Frequency for the variables in the study for the whole, Swedish, and Polish samples.

		All students (N=646), n (%)	Swedish students (n_Sweden_=342), n (%)	Polish students (n_Poland_=304), n (%)
**Age (years)**				
	18-25	478 (73.9)	179 (52.3)	299 (98.4)
	>25	168 (26)	163 (47.7)	5 (1.6)
**Gender**				
	Women	555 (85.9)	284 (83)	271 (89.1)
	Men	89 (13.8)	56 (16.4)	33 (10.9)
**Semester**				
	1	289 (44.7)	158 (46.2)	131 (43.1)
	3	208 (32.2)	101 (29.5)	107 (35.2)
	5	149 (23.1)	83 (24.2)	66 (21.7)
**eHEALS^a^ score**				
	<3	43 (6.7)	23 (6.7)	24 (7.9)
	≥3	603 (93.3)	319 (93.3)	284 (93.4)
**High school focus**				
	Health and social care	235 (36.4)	82 (23.9)	153 (50.3)
	Technology	25 (3.9)	11 (3.2)	14 (4.6)
	Other	374 (57.9)	242 (70.8)	132 (43.4)
**Previous work experience**				
	Health and social care	211 (32.7)	188 (54.9)	23 (7.6)
	Technology	12 (1.9)	7 (2)	5 (1.6)
	Other	332 (51.4)	118 (34.5)	214 (70.4)
**Skills: computer**				
	1: not knowledgeable at all	1 (0.2)	0 (0)	1 (0.3)
	2	19 (2.9)	14 (4)	5 (1.6)
	3	134 (20.7)	93 (27.2)	41 (13.5)
	4	173 (26.8)	106 (30.9)	67 (22)
	5: very knowledgeable	306 (47.4)	116 (33.9)	190 (62.5)
**Skills: smartphone**				
	1: not knowledgeable at all	4 (0.6)	3 (0.9)	1 (0.3)
	2	5 (0.8)	3 (0.9)	2 (0.7)
	3	6 (0.9)	41 (11.9)	22 (7.2)
	4	127 (19.7)	88 (25.7)	39 (12.9)
	5: very knowledgeable	447 (69.2)	207 (60.5)	240 (78.9)
**Skills: tablets**				
	1: not knowledgeable at all	66 (10.2)	25 (7.3)	41 (13.5)
	2	69 (10.7)	47 (13.7)	22 (7.2)
	3	107 (16.6)	66 (19.3)	41 (13.5)
	4	149 (23.1)	88 (25.7)	61 (20)
	5: very knowledgeable	239 (36.9)	100 (29.2)	139 (45.7)
**Frequency: computer**				
	Several times daily	131 (20.3)	69 (20.2)	62 (20.4)
	Daily	183 (28.3)	80 (23.4)	103 (33.9)
	Every week	152 (23.5)	85 (24.9)	67 (22)
	Every month	39 (6)	29 (8.5)	10 (3.3)
	Sometimes	98 (15.2)	42 (12.3)	56 (18.4)
	Never	11 (1.7)	5 (1.5)	6 (1.9)
**Frequency: smartphone**				
	Several times daily	576 (89.2)	302 (88.3)	274 (90.1)
	Daily	62 (9.6)	34 (9.9)	28 (9.2)
	Every week	3 (0.5)	2 (0.6)	1 (0.3)
	Every month	0 (0)	0 (0)	0 (0)
	Sometimes	2 (0.3)	1 (0.3)	1 (0.3)
	Never	1 (0.2)	1 (0.3)	0 (0)
**Frequency: tablet**				
	Several times daily	29 (4.5)	23 (6.7)	6 (1.9)
	Daily	43 (6.7)	26 (7.6)	17 (5.6)
	Every week	51 (7.9)	38 (11.1)	13 (4.3)
	Every month	23 (3.6)	14 (4.1)	9 (2.9)
	Sometimes	138 (21.4)	76 (22.2)	62 (20.4)
	Never	332 (51.4)	135 (39.5)	197 (64.8)

^a^eHEALS: eHealth Literacy Scale.

The mean and SD values for the self-reported techEnthusiasm for the whole sample, Swedish students, and Polish students were 3.83 (SD 0.90), 3.62 (SD 0.94), and 4.04 (SD 0.78), respectively, which constitutes a high overall technophilia. On the other hand, the mean and SD values for techAnxiety for the whole sample, Swedish students, and Polish students were 2.48 (SD 0.96), 2.37 (SD 1), and 2.60 (SD 0.89), respectively, displaying midrange values regarding the negative feelings toward technology.

Multimedia Appendix 2 shows the mean and SD values for both techEnthusiasm and techAnxiety according to different levels of socioeconomic, eHEALS, perceived skill, and frequency variables. The association of these variables with techEnthusiasm and techAnxiety is investigated in the following sections.

### Factors Associated With TechEnthusiasm

The Spearman rank correlation coefficient was used to investigate the association of techEnthusiasm and the nonparametric variables of ordinal scale in the study, namely, age, semester, eHEALS score, perceived skill, and frequency of using electronic devices. These results are shown in terms of the Swedish, Polish, and overall samples in Table 2.

**Table 2:** Spearman rank correlation coefficient calculated for the whole sample, Swedish students, and Polish students separately, regarding technology enthusiasm.

	All students		Swedish students			Polish students	
	*P* value	ρ (95% CI)	*P* value	ρ (95% CI)	*P* value		ρ (95% CI)
Age	<.001	−0.238 (−0.310 to −0.163)	<.001	−0.201 (−0.302 to −0.096)	.65		−0.027 (−0.139 to 0.086)
Semester	.96	0.002 (−0.075 to 0.079)	.53	−0.034 (−0.140 to 0.073)	.44		0.044 (−0.068 to 0.156)
eHEALS^a^ score	<.001	0.265 (0.190 to 0.336)	<.001	0.190 (0.084 to 0.291)	<.001		0.352 (0.246 to 0.449)
Skill: computer	<.001	0.360 (0.288 to 0.428)	<.001	0.341 (0.238 to 0.436)	<.001		0.309 (0.201 to 0.410)
Skill: smartphone	<.001	0.385 (0.315 to 0.452)	<.001	0.352 (0.253 to 0.445)	<.001		0.364 (0.258 to 0.460)
Skill: tablet	<.001	0.269 (0.194 to 0.342)	<.001	0.309 (0.204 to 0.406)	<.001		0.204 (0.092 to 0.310)
Frequency: computer	<.001	−0.153 (−0.230 to −0.074)	<.001	−0.176 (−0.283 to −0.065)	.01		−0.146 (−0.255 to −0.034)
Frequency: smartphone	.95	0.002 (−0.075 to 0.080)	.97	−0.002 (−0.109 to 0.105)	.87		0.009 (−0.103 to 0.122)
Frequency: tablet	.92	0.004 (−0.075 to 0.083)	.16	−0.079 (−0.189 to 0.032)	.59		−0.031 (−0.143 to 0.081)

^a^eHEALS: eHealth Literacy Scale.

A negative correlation was found between age and techEnthusiasm for the Swedish sample and overall sample, indicating that greater the age, lesser the techEnthusiasm score (*P*<.001; ρ_all_=−0.238; ρ_Swedish_=−0.201). This association might not have been significant for the Polish sample due to the lack of age variance observed in the Swedish sample (refer to Figure 1—the Polish students’ age distribution presents a heavier tail compared to the Swedish ones). A positive correlation was found between eHealth literacy and techEnthusiasm, indicating that greater the eHEALS score, greater the techEnthusiasm score (*P*<.001; ρ_all_=0.265; ρ_Swedish_=0.190; ρ_Polish_=0.352). A positive correlation was found between perceived skill in all investigated electronic devices and techEnthusiasm, indicating that greater the perceived skill, greater the techEnthusiasm (Table 2). In terms of frequency of use, a negative correlation was found between the use of computers and techEnthusiasm (*P*<.001 ρ_all_=−0.153; ρ_Swedish_=−0.176; ρ_Polish_=−0.146). The negative values of ρ are due to the inverted Likert scale used for the question, that is, from “several times daily” to “never.” Thus, the techEnthusiasm score increases with higher frequencies of use. It is noteworthy that even with low ρ values, the significant associations found are still relevant due to the large sample size. The narrow 95% CIs indicate low variability and stability of the results.

The Mann-Whitney *U* test was used to assess gender differences regarding the students’ reported techEnthusiasm. No significant differences were found for Swedish, Polish, or overall samples (*P*_Swedish_=.45, *P*_Polish_=.38, and *P*_all_=.68).

### Factors Associated With TechAnxiety

Analogous statistical analyses were performed on the techAnxiety scores for the Swedish, Polish, and overall samples. Table 3 shows the Spearman rank correlation coefficients calculated for the same variables as for techEnthusiasm. A positive correlation was found between age and techAnxiety in the Swedish sample, indicating that greater the age, greater the techAnxiety score (*P*<.05; ρ_Swedish_=0.184). Similar to techEnthusiasm, this association might not have been significant for the Polish sample due to the lack of age variance (Figure 1). A negative correlation was found between higher semesters and techAnxiety in the Swedish and overall samples, indicating that higher the students were in their education, lesser the techAnxiety score (*P*<.05; ρ_all_=−0.101; ρ_Swedish_=−0.124).

**Table 3:** Spearman rank correlation coefficient calculated for the whole sample, Swedish students, and Polish students separately, regarding technology anxiety.

Feature	All students		Swedish students			Polish students	
	*P* value	ρ (95% CI)	*P* value	ρ (95% CI)	*P* value		ρ (95% CI)
Age	.54	0.024 (−0.101 to 0.053)	<.001	0.184 (0.078 to 0.286)	.45		−0.043 (−0.155 to 0.070)
Semester	.01	−0.101 (−0.178 to −0.024)	.02	−0.124 (−0.229 to −0.018)	.19		−0.075 (−0.186 to 0.038)
eHEALS^a^ score	<.001	−0.196 (−0.270 to −0.120)	<.001	−0.262 (−0.360 to −0.158)	.02		−0.133 (−0.242 to −0.020)
Skill: computer	<.001	−0.209 (−0.283 to −0.132)	<.001	−0.347 (−0.442 to −0.245)	.02		−0.134 (−0.244 to −0.022)
Skill: smartphone	<.001	−0.165 (−0.240 to −0.088)	<.001	−0.245 (−0.345 to −0.141)	.046		−0.114 (−0.224 to −0.002)
Skill: tablet	<.001	−0.251 (−0.324 to −0.175)	<.001	−0.347 (−0.442 to −0.244)	<.001		−0.191 (−0.298 to −0.080)
Frequency: computer	.495	0.028 (−0.052 to 0.107)	.16	0.080 (−0.033 to 0.191)	.61		−0.029 (−0.142 to 0.083)
Frequency: smartphone	.17	0.055 (−0.023 to 0.132)	.27	0.060 (−0.047 to 0.166)	.37		0.052 (−0.061 to 0.164)
Frequency: tablet	.19	0.053 (−0.026 to 0.132)	.77	0.017 (−0.095 to 0.128)	.50		0.039 (−0.074 to 0.151)

^a^eHEALS: eHealth Literacy Scale.

A negative correlation was found between eHealth literacy and techAnxiety (*P*<.001; ρ_all_=−0.196; ρ_Swedish_=−0.262; ρ_Polish_=−0.133). A negative correlation was found between the perceived skill in all investigated devices and techAnxiety, indicating that greater the perceived skill, lesser the techAnxiety score (Table 3). Similar to techEnthusiasm, the low ρ values still show relevant associations due to the sample size. In addition, similar to techEnthusiasm, the narrow 95% CIs indicate low variability and stability of the results.

**Figure 2:**
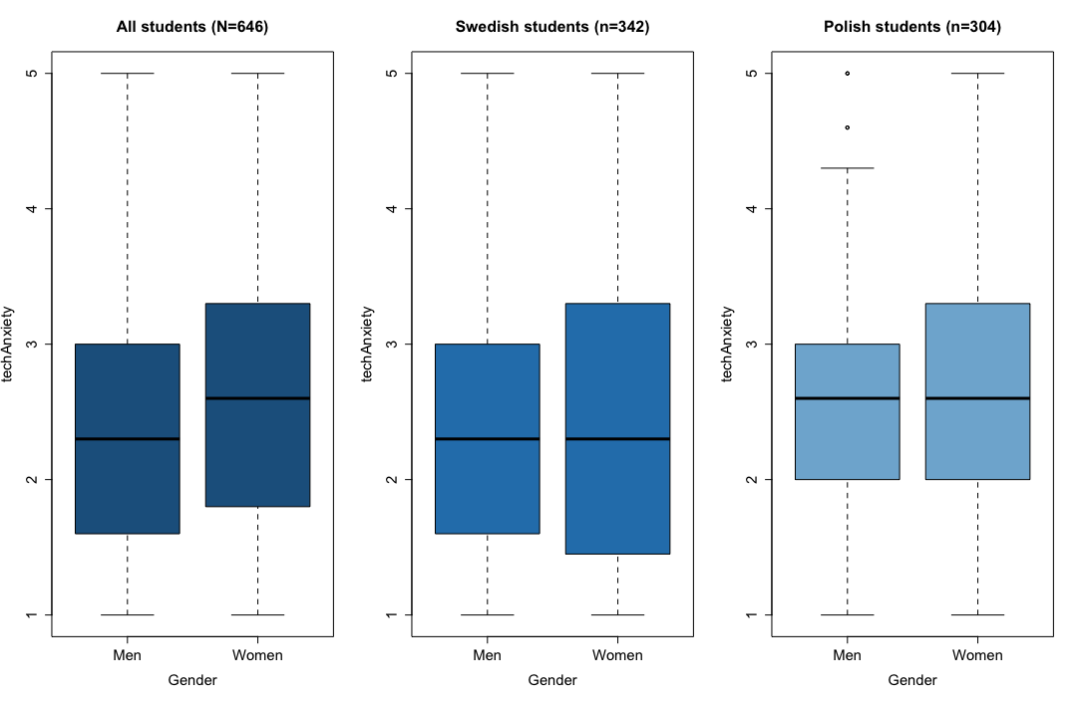
Box plots for the reported technology anxiety in the whole, Swedish, and Polish samples.

Gender differences regarding the students’ reported techAnxiety were observed through the Mann-Whitney *U* test for the whole sample and the Swedish students (*P*_Swedish_=.002; *P*_Polish_=.69; *P*_all_=.01). A considerable difference in the mean scores of the reported techAnxiety can be observed between men (1.987, SD 0.854) and women (2.451, SD 1.014) in the Swedish sample of students. This was not observed for the whole sample, with means of 2.240 (SD 0.90) and 2.521 (SD 0.963) for men and women students, respectively. However, upon closer inspection of the boxplots shown in Figure 2, the attributed gender differences can be observed when the distribution is analyzed. The Swedish women students present a higher dispersion of techAnxiety scores, whereas men present a heavier tail distribution, which in turn increases the distance between these groups.

## Discussion

### Principal Findings

#### Overview

This cross-sectional, multicenter study aimed to determine Swedish and Polish students’ attitudes toward technology, specifically directed to enthusiasm and anxiety, and factors associated with those attitudes. The principal findings of this study are as follows: (1) in the Swedish sample (mean age 27, SD 7.34 years), the older the students were, the more anxious and less enthusiastic they were about technology; (2) the higher the students’ eHealth literacy score was, the more enthusiastic and less anxious they were regarding technology (both Swedish and Polish samples); (3) the higher the perceived skill in using electronic devices was, the more enthusiastic and less anxious about technology the students were (both Swedish and Polish samples); (4) in the Swedish sample, the more senior the students were in their education (higher semesters), the less anxious they were toward technology they were; and (5) gender differences were found in the Swedish sample regarding anxiety toward technology. These will be further discussed in the following sections.

#### Attitudes Toward Technology and Age

The positive correlation between age and techAnxiety and negative correlation between age and techEnthusiasm, meaning that greater the age, lesser the enthusiasm and higher the anxiety toward technology, is an interesting finding, as many Swedish students (163/342, 47.6%) fall into the mature student category. This concept does not have a definition, but published literature usually considers the individuals who enter higher education at the age of 26 to 30 as mature students who are believed to be different from their younger colleagues [29]. The fact that many universities have a changing cohort with a higher rate of accepted mature students, meanwhile adopting more and more technologies as teaching enhancements [30], raises concerns about how the students’ attitudes toward technology could affect their learning. Technology-enhanced learning methods in the classroom can promote high-order thinking, that is, rationalizing on a level higher than memorizing or telling facts as told [31]. These teaching approaches affect the attainment of the subject being taught and decrease subject anxieties [29,32,33]. In the specific case of nursing, a systematic review of literature by Labrague et al [34] shows positive results in using high-fidelity simulations for enhancing the self-confidence of nursing students in managing their duties. Identifying the students’ needs could be important in these scenarios, so that learning could be efficiently delivered. A recent study investigated mature students’ attitudes toward technology and found no significant differences from younger students. However, the attitudes considered in the study instrument were confidence and a sense of utility [29]. The hypothesis suggests that the observed phenomenon related to age may not be applicable to the Polish sample due to its younger ages and lower variability (mean 20.4, SD 1.72 years).

#### Attitudes Toward Technology and eHealth Literacy

The positive correlation between eHealth literacy score and enthusiasm toward technology is not surprising because computer literacy is one of the domains assessed in the eHEALS instrument. The negative correlation between eHealth literacy score and anxiety toward technology is also important to be considered. Even with different approaches to eHealth in nursing education, it seems important for the nursing students’ attitudes toward technology, which would later influence their use of technology in clinical scenarios. Registered nurses commonly rely on advice from their colleagues as their primary information source to inform daily clinical practice [35-37]. However, this information channel has an inherent risk of diverging from the best evidence available in published literature, which could impact the quality of patient care [38]. The best clinical practices can be readily accessed through reference materials and web-based publications in nursing journals. Thus, if anxiety toward technology is a factor that is identified as a barrier to pursuing such information, this means that it should be addressed in their education.

#### Attitudes Toward Technology and Perceived Skills in Using Electronic Devices

Another study finding suggests that students exhibiting higher perceived skill in using electronic devices (computers or laptops, smartphones, and tablets) also demonstrated more enthusiastic and less anxious attitudes toward technology. Only few studies have investigated electronic device use in nursing education. However, investigating this topic is important because despite nursing students reporting proficiency in computer skills, a lack of exposure to new devices can still lead to hesitancy in their use [39]. In the recent years, mobile apps have been trialed and shown to support the education and practice training of nursing students [40]. This technology facilitates access to patient care resources, fostering self-directed learning and problem-solving [41]. A study by Kenny et al [42] investigated the impact of using smartphones and tablets with a QR code scanning app linking to educational information on the nursing students’ anxiety levels while performing psychomotor skills in the patient care setting. The study found that providing students with access to these tools helped to reduce anxiety by offering quick access to reputable patient care information [42]. Previous studies of bank employees also found an inverse relationship between techAnxiety and computer skills [43]. The study did not find any significant associations with the frequency of use, which is consistent with this study, with the exception of techEnthusiasm related to computer or notebook. However, it can be argued that this could simply be a direct result of being enthusiastic and wanting to engage with it daily. While no studies in the literature approached the topic of techEnthusiasm and skill, a study by Revilla Muñoz et al [44] reported lower levels of techAnxiety in high school teachers after information and communications technology training.

#### Attitudes Toward Technology and Semester

In the Swedish sample, students exhibited lower levels of anxiety toward technology the further in their education they were. This could be related to how health technology topics are being addressed in specific courses given in higher semesters, which was not the case for the Polish university at the time of the study. This could indicate that having specific courses with eHealth and health technology curricula could be useful to address techAnxiety in students. A scoping review by Nes et al [45] highlights that the current state of nursing education indicates a prevalent lack of focus on technology and technological literacy, favoring teaching over engaging with technological advancements in the clinical field, resulting in limited exposure to such developments. This holds significance because practitioners are likely to navigate ongoing technological advancements throughout their careers. Therefore, nursing education should be viewed as a platform that fosters lifelong learning, placing emphasis on proactive engagement and critical thinking in response to technological progress [46].

#### Attitudes Toward Technology and Gender

This study also found gender differences regarding techAnxiety in the Swedish students (mature student sample). There is sparse published literature about gender and computer anxiety, and findings do not seem to provide a conclusion [47-50]. Sparse literature has been published in the area of techAnxiety and even less so in the techEnthusiasm domain; this may compromise the credibility of the findings from comparing these studies. It can be argued that the findings of studies conducted more than a decade ago are difficult to interpret without the context of the time they were published in, because with the rapid technological advancements of the past years, the relationship between users and technology has changed drastically.

### Implications to Practice

Understanding the factors that influence techEnthusiasm and techAnxiety holds important practical implications, particularly in the context of health care innovation and access to care.

TechAnxiety and techEnthusiasm can impact the technology acceptance level of a new health care solution. Low levels of acceptance are related to implementation delays and even complete system failures [51]. According to the systematic literature review by AlQudah et al [52], which included 142 studies, the key factors associated with health technology acceptance are its ease of use and perceived usefulness, which are measured using the widespread Technology Acceptance Model instrument. In addition, anxiety and computer self-efficacy are the next extensively studied factors related to health care technology acceptance, which aligns with the focus of this study.

A qualitative study conducted with nurses who have lower levels of digital literacy [53] explored factors related to health IT acceptance in this population. The results portrayed that these nurses show little enthusiasm toward technology and even considered the use of such technological tools as “bad patient-centered care.” Addressing those attitudes toward technology is a challenge and should be tailored to special needs, as these individuals also reported that the training sessions are conducted in large groups and that the pace is too fast for them.

Telemedicine, eHealth records, health IT systems, and mobile apps emerge as important health technologies that are directed to improve productivity and effectiveness of the health care sector. During the COVID-19 pandemic, digital health strategies, which include such systems, were imperative for providing continuity of care; economic, social, geographical, time, and cultural accessibility; and coordination of care, among others [54]. However, during those difficult times, several health professionals were unprepared to use such technologies [54]. Having health personnel that is trained to use different health technologies proved to be imperative to build preparedness for unusual health emergency situations. Strategies to address the problem of accessibility of health care in remote or rural areas could also use such technologies [52]. Hence, it is important to understand students’ digital savviness to devise strategies to address health technology topics accordingly in the curricula of health-related undergraduate programs.

### Limitations

As this was a self-reported survey study, care must be taken when extrapolating the results shown in this paper. Response bias was mitigated by involving researchers with no educational connections with the surveyed participants. An earlier study of self-reported technology use presented only marginal errors to the respondents’ true use [55]. Another important limitation of this study is the disproportionate number of women and men participants, with the former consisting of 85.9% (555/646) of the whole sample. Although the statistical tests used in the analyses are robust against data imbalance, the magnitude of such imbalance could have affected the results. In addition, the use of a convenience sample can limit how the findings of this study can be generalized. However, in this study, the involvement of different universities from different countries as data sources helped to reduce this risk. Finally, the instrument used in this study was initially crafted and validated for use with older adults. Although published evidence exists for its use in younger populations [23,24], the results should be interpreted with caution, recognizing the potential for age-related bias.

### Conclusions

This cross-sectional, multicenter study emphasized the importance of nursing students’ enthusiasm and anxiety toward technology and highlighted the factors associated with these attitudes. As health care increasingly relies on technologies such as telemedicine, eHealth records, health IT systems, and mobile apps, the integration of health technology topics into educational curricula becomes imperative, taking the students’ attitudes toward technology into consideration, so that in the future, these professionals are prepared to address future health care challenges. Future qualitative studies should investigate nursing students who portray high anxiety and low enthusiasm toward technology to further validate the results presented in this paper and understand their points of view, so that pedagogical strategies can be developed to incorporate health technology topics in the curricula.

## Appendix

Multimedia Appendix 1 STROBE (Strengthening the Reporting of Observational studies in Epidemiology) statement—checklist of items that should be included in the reports of cross-sectional studies.

Multimedia Appendix 2 Descriptive statistics in terms of sociodemographic variables, technology anxiety, and technology enthusiasm. The first panel shows the frequency and percentages, and the second and third panels show the results in terms of the technology anxiety and technology enthusiasm scores.

## Abbreviations

BTH: Blekinge Institute of Technology
ECTS: European Credit Transfer and Accumulation System
eHEALS: eHealth Literacy Scale
MUB: Medical University of Bialystok
SRCU: Swedish Red Cross University
STROBE: Strengthening the Reporting of Observational studies in Epidemiology
TechAnxiety: technology anxiety
TechEnthusiasm: technology enthusiasm
TechPH: Technophilia instrument

## Data Availability

The data sets generated during and analyzed during this study are available from the corresponding author on reasonable request.
