# Supplementary material for: Nursing Students’ Attitudes Toward Technology: Multicenter Cross-Sectional Study
Source: JMIR Med Educ. 2024 Apr 29;10:e50297. doi: 10.2196/50297 (PMC11091804; doi:10.2196/50297)
Supplement: Multimedia Appendix 2 [file mededu_v10i1e50297_app2.pdf]

Multimedia appendix 2

Multimedia appendix 2 table. Descriptive statistics in terms of sociodemographic variables, techAnxiety and techEnthusiasm. The first panel shows the frequency and percentages, the second and third panels show the results in terms of the techAnxiety and techEnthusiasm scores.

Characteristic	Frequency (%)			techAnxiety mean (SD)			techEnthusiasm mean (SD)		
	All	Sweden	Poland	All	Sweden	Poland	All	Sweden	Poland
**Age**									
18-25 years	478(74)	179(27.7)	299(46.3)	2.5(0.9)	2.2(0.9)	2.6(0.9)	4.0(0.8)	3.8(0.9)	4.1(0.8)
> 25 years	168(26)	163(25.2)	5(0.8)	2.5(1.0)	2.5(1.0)	2.5(0.9)	3.5(1.0)	3.5(1.0)	3.4(0.8)
**Gender**									
Female	555(85.9)	284(44.0)	271(42.0)	2.5(1.0)	2.5(1.0)	2.6(0.9)	3.8(0.9)	3.6(0.9)	4.1(0.8)
Male	89(13.8)	56(8.7)	33(5.1)	2.2(0.9)	2(0.9)	2.7(0.8)	3.8(0.8)	3.7(0.9)	4.0(0.7)
**Semester**									
1	289(44.7)	158(24.5)	131(20.3)	2.6(1.0)	2.5(1.0)	2.7(0.9)	3.8(0.9)	3.6(1.0)	4(0.8)
3	208(32.2)	101(15.6)	107(16.6)	2.4(1.0)	2.2(1.0)	2.6(0.9)	3.9(0.9)	3.7(1.0)	4(0.8)
5	149(23.1)	83(12.8)	66(10.2)	2.4(0.9)	2.3(0.9)	2.5(1.0)	3.8(0.9)	3.5(0.9)	4.2(0.7)
**eHeals**									
**< 3**	43 (6.7)	23(3.6)	24(3.3)	2.9(0.96)	2.7(0.9)	3.0(1.0)	3.4(0.9)	3.3(0.9)	3.5(0.9)
**≥ 3**	603 (93.3)	319 (49.3)	284 (43.8)	2.4(1.0)	2.3(1.0)	2.5(0.9)	3.9(0.9)	3.6(0.9)	4.1(0.8)
**High school focus**									
Health and social care	235(36.4)	82(12.7)	153(23.7)	2.6(1.0)	2.5(1.1)	2.6(1.0)	4.0(0.8)	3.8(0.9)	4.1(0.8)
Technology	25(3.9)	11(1.7)	14(2.2)	2.6(1.2)	2.4(1.3)	2.7(1.1)	3.8(0.7)	3.5(0.8)	4.1(0.6)
Other	374(57.9)	242(37.5)	132(20.4)	2.4(0.9)	2.3(1.0)	2.6(0.8)	3.7(0.9)	3.6(1.0)	4.0(0.8)
**Previous work experience**									
Health and social care	211(32.7)	188(29.1)	23(2.6)	2.4(0.9)	2.4(1.0)	2.8(0.9)	3.8(0.9)	3.8(0.9)	4.1(0.7)
Technology	12(1.9)	7(1.1)	5(0.8)	2.6(0.7)	2.3(0.6)	3.0(0.8)	4.0(0.8)	3.8(0.7)	4.3(0.8)
Other	332(51.4)	118(18.3)	214(33.1)	2.5(0.9)	2.4(1.0)	2.6(0.9)	3.8(0.9)	3.4(1.0)	4.1(0.8)
**Skills - computer**									
Not knowledgeable at all - 1	1(0.2)	0(0)	1(0.2)	2.6(-)	NA(-)	2.6(-)	3(-)	NA(-)	3(-)
2	19(2.9)	14(2.2)	5(0.8)	3.2(1.0)	3.2(1.0)	3.2(0.9)	3.1(1.1)	2.9(1.1)	3.8(0.9)
3	134(20.7)	93(14.4)	41(6.3)	2.8(0.9)	2.7(1.0)	2.8(0.7)	3.4(1.0)	3.2(1.0)	3.7(0.9)
4	173(26.8)	106(16.4)	67(10.4)	2.5(0.9)	2.4(1.0)	2.6(0.8)	3.7(0.8)	3.7(0.8)	3.8(0.7)
Very knowledgeable - 5	306(47.4)	116(18)	190(29.4)	2.3(1.0)	2(0.9)	2.5(1.0)	4.1(0.8)	4(0.8)	4.2(0.7)
**Skills – smartphone**									
Not knowledgeable at all - 1	4(0.6)	3(0.5)	1(0.2)	3(0.4)	3.2(0.3)	2.6(-)	2.9(1.1)	2.9(1.4)	3(-)
2	5(0.8)	3(0.5)	2(0.3)	3.4(0.8)	3.8(0.7)	2.8(0.3)	2.9(1.0)	2.3(0.7)	3.8(0.3)
3	63(9.8)	41(6.3)	22(3.4)	2.8(0.8)	2.8(1.0)	2.9(0.5)	3.1(0.9)	2.9(1.0)	3.4(0.8)
4	127(19.7)	88(13.6)	39(6)	2.6(0.9)	2.5(1.0)	2.7(0.8)	3.5(0.7)	3.5(0.8)	3.7(0.7)
Very knowledgeable - 5	447(69.2)	207(32)	240(37.2)	2.4(1.0)	2.2(1.0)	2.6(0.9)	4(0.8)	3.9(0.9)	4.2(0.7)

**Skills - tablets**									
Not knowledgeable at all - 1	66(10.2)	25(3.9)	41(6.3)	2.8(0.9)	2.9(1.0)	2.8(0.9)	3.7(1.0)	3.3(1.1)	4(0.9)
2	69(10.7)	47(7.3)	22(3.4)	2.8(0.9)	2.8(1.0)	2.9(0.6)	3.5(0.8)	3.4(0.8)	3.8(0.6)
3	107(16.6)	66(10.2)	41(6.3)	2.7(0.8)	2.7(0.9)	2.7(0.8)	3.5(1.0)	3.2(1.0)	3.9(0.8)
4	149(23.1)	88(13.6)	61(9.4)	2.4(1.0)	2.3(1.0)	2.7(0.9)	3.8(0.8)	3.8(0.8)	3.9(0.8)
Very knowledgeable - 5	239(37.0)	100(15.5)	139(21.5)	2.2(1.0)	1.9(0.9)	2.4(0.9)	4.2(0.8)	4(0.8)	4.2(0.7)
**Frequency - computer**									
Several times daily	131(20.3)	69(10.7)	62(9.6)	2.5(1.0)	2.4(1.1)	2.6(0.9)	4.1(0.8)	3.9(0.8)	4.3(0.7)
Daily	183(28.3)	80(12.4)	103(15.9)	2.5(0.9)	2.3(1.0)	2.7(0.9)	3.9(0.8)	3.6(0.9)	4.1(0.8)
Every week	152(23.5)	85(13.2)	67(10.4)	2.4(0.9)	2.3(0.9)	2.6(1.0)	3.6(1.0)	3.4(1.0)	3.9(0.8)
Every month	39(6.0)	29(4.5)	10(1.5)	2.6(1.2)	2.6(1.3)	2.6(0.9)	3.6(0.9)	3.5(1.0)	3.9(0.7)
Sometimes	98(15.2)	42(6.5)	56(8.7)	2.5(0.9)	2.6(1.0)	2.5(0.8)	3.8(1.0)	3.6(1.1)	4(0.8)
Never	11(1.7)	5(0.8)	6(0.9)	2.9(0.8)	2.8(0.8)	3(1.0)	3.3(0.8)	2.8(0.6)	3.8(0.7)
**Frequency - smartphones**									
Several times daily	576(89.2)	302(46.7)	274(42.4)	2.5(1.0)	2.4(1.0)	2.6(0.9)	3.8(0.9)	3.6(0.9)	4.1(0.8)
Daily	62(9.6)	34(5.3)	28(4.3)	2.6(1.0)	2.5(1.1)	2.7(0.9)	3.9(1.0)	3.7(1.1)	4.2(0.7)
Every week	3(0.5)	2(0.3)	1(0.2)	3.1(1.3)	3.4(1.6)	2.3(NA)	2.3(0.7)	2.3(1.0)	2.3(NA)
Every month	0(0)	0(0)	0(0)	NA(-)	NA(-)	NA(-)	NA(-)	NA(-)	NA(-)
Sometimes	2(0.3)	1(0.2)	1(0.2)	2.5(0.2)	2.3(-)	2.6(-)	3.5(0.2)	3.6(-)	3.3(-)
Never	1(0.2)	1(0.2)	0(0)	3.6(-)	3.6(-)	NA(-)	1.3(-)	1.3(-)	NA(-)
**Frequency - tablets**									
Several times daily	29(4.5)	23(3.6)	6(0.9)	2.5(1.2)	2.4(1.2)	2.5(1.2)	4.2(0.8)	4.1(0.8)	4.6(0.7)
Daily	43(6.7)	26(4.0)	17(2.6)	2.4(1.0)	2.3(1.2)	2.6(0.8)	3.8(0.8)	3.7(0.7)	3.9(0.9)
Every week	51(7.9)	38(5.9)	13(2.0)	2.4(1.0)	2.4(1.0)	2.5(0.9)	3.8(0.8)	3.6(0.8)	4.3(0.5)
Every month	23(3.6)	14(2.2)	9(1.4)	2.4(1.0)	2.3(1.2)	2.5(0.8)	3.8(0.9)	3.4(0.9)	4.3(0.5)
Sometimes	138(21.4)	76(11.8)	62(9.6)	2.4(1.0)	2.3(1.0)	2.6(1.0)	3.8(0.9)	3.7(0.9)	4(0.9)
Never	332(51.4)	135(20.9)	197(30.5)	2.5(0.9)	2.4(1.0)	2.6(0.9)	3.8(0.9)	3.5(1.1)	4.1(0.8)

Acronyms: NA: Not Applicable; SD: Standard deviation
